# Exogenous Hydrogen Sulfide Regulates the Growth of Human Thyroid Carcinoma Cells

**DOI:** 10.1155/2019/6927298

**Published:** 2019-05-16

**Authors:** Dongdong Wu, Jianmei Li, Qianqian Zhang, Wenke Tian, Peiyu Zhong, Zhengguo Liu, Huijuan Wang, Honggang Wang, Ailing Ji, Yanzhang Li

**Affiliations:** ^1^School of Basic Medical Sciences, Henan University, Kaifeng, Henan 475004, China; ^2^Henan International Joint Laboratory for Nuclear Protein Regulation, Henan University, Kaifeng, Henan 475004, China

## Abstract

Hydrogen sulfide (H_2_S) is involved in the development and progression of many types of cancer. However, the effect and mechanism of H_2_S on the growth of human thyroid carcinoma cells remain unknown. In the present study, we found that the proliferation, viability, migration, and invasion of human thyroid carcinoma cells were enhanced by 25–50 *μ*M NaHS (an H_2_S donor) and inhibited by 200 *μ*M NaHS. However, H_2_S showed no obvious effects on the proliferation, viability, and migration of human normal thyroid cells. Administration of 50 *μ*M NaHS increased the expression levels of CBS, SQR, and TST, while 200 *μ*M NaHS showed reverse effects in human thyroid carcinoma cells. After treatment with 25-50 *μ*M NaHS, the ROS levels were decreased and the protein levels of p-PI3K, p-AKT, p-mTOR, H-RAS, p-RAF, p-MEK1/2, and p-ERK1/2 were increased, whereas 200 *μ*M NaHS exerted opposite effects in human thyroid carcinoma cells. Furthermore, 1.4-2.8 mg/kg/day NaHS promoted the tumor growth and blood vessel formation in human thyroid carcinoma xenograft tumors, while 11.2 mg/kg/day NaHS inhibited the tumor growth and angiogenesis. In conclusion, our results demonstrate that exogenous H_2_S regulates the growth of human thyroid carcinoma cells through ROS/PI3K/Akt/mTOR and RAS/RAF/MEK/ERK signaling pathways. Novel H_2_S-releasing donors/drugs can be designed and applied for the treatment of thyroid cancer.

## 1. Introduction

Hydrogen sulfide (H_2_S) is the third member of the gasotransmitter family along with nitric oxide and carbon monoxide [1–3]. H_2_S can be endogenously produced from L-cysteine (L-Cys) and homocysteine in mammalian tissues mainly by two pyridoxal-5′-phosphate- (PLP-) dependent enzymes, cystathionine *β*-synthase (CBS) and cystathionine *γ*-lyase (CSE). CBS and CSE are predominantly cytosolic enzymes [4–6]. 3-Mercaptopyruvate sulfurtransferase (3-MST) is a PLP-independent enzyme that acts in combination with cysteine aminotransferase (CAT) to produce H_2_S from L-Cys in the presence of *α*-ketoglutarate. 3-MST and CAT are located in the cytosol and mitochondria [6–8]. In addition, D-amino acid oxidase metabolizes D-cysteine to an achiral *α*-ketoacid, 3-mercaptopyruvate, which can be further metabolized to H_2_S by 3-MST in both the brain and the kidney [9]. H_2_S can be immediately released or stored in the forms of bound sulfane sulfur and acid-labile sulfur in the cells [10–13].

There is increasing evidence that H_2_S plays important roles in a number of physiological conditions, including angiogenesis [14], vascular relaxation [15], neuronal activity [16], energy production [17], and glucose regulation [18]. However, abnormal H_2_S metabolism is associated with many diseases, such as atherosclerosis [19], diabetes [20], asthma [21], hypertension [22], and neurodegenerative diseases [23]. Thyroid cancer is the most common endocrine-related cancer with a rapid worldwide rise in incidence in the past few decades [24]. The prognosis of thyroid cancer is correlated with the progression of localized primary tumors to advanced stages, which ultimately metastasizes to multiple organs [25]. It has been shown that H_2_S is involved in the development and progression of many different types of cancer [5, 26–28]. However, the effect and mechanism of H_2_S on the growth of human thyroid carcinoma cells remain unknown.

In this study, we investigated the effect and mechanism of H_2_S on the proliferation, viability, migration, and invasion of human thyroid carcinoma cells. We further examined the effects of H_2_S on tumor growth and angiogenesis in nude mice xenografted with human thyroid carcinoma.

## 2. Materials and Methods

### 2.1. Cell Culture

Human normal thyroid cell line Nthy-ori3-1 and human thyroid carcinoma cell lines TPC-1, TT, and ARO were obtained from CoBioer Biosciences Co. Ltd. (Nanjing, Jiangsu, China). Cells were cultured in RPMI 1640 medium supplemented with 10% fetal bovine serum, 100 units/ml penicillin, and 100 *μ*g/ml streptomycin. Cells were grown at 37°C in a humidified atmosphere of 95% air and 5% CO_2_ [29]. Confluent cells were starved overnight in a serum-free RPMI 1640 medium. The cells were then treated with 10, 25, 50, 100, and 200 *μ*M NaHS (an H_2_S donor, Sigma-Aldrich, St. Louis, MO, USA) for 24 h. The phosphate-buffered saline (PBS) group was served as a control.

### 2.2. 5-Ethynyl-2′-Deoxyuridine (EdU) Assay

Cell proliferation ability was detected by the Cell-Light EdU Apollo 567 *In Vitro* Imaging Kit (RiboBio, Guangzhou, Guangdong, China). Cell proliferation rate = (number of EdU − positive cells)/(total number of cells) × 100% [30].

### 2.3. MTS Assay

The cell viability was detected using the CellTiter 96 AQ_ueous_ One Solution Cell Proliferation Assay kit (MTS; Promega, Madison, WI, USA).

### 2.4. Cell Counting Kit-8 (CCK-8) Assay

The CCK-8 detection kit (Beyotime Institute of Biotechnology, Shanghai, China) was used to measure cell viability.

### 2.5. Wound Healing Assay

Cultured cells in confluent monolayer were wounded using a sterile micropipette tip. Cell migration was observed under an Olympus CKX41 microscope and measured using the ImageJ software (National Institute for Health, Bethesda, MD, USA). The migration rate (MR) was calculated according to the formula: MR = [(*A* − *B*)/*A*] × 100%, where *A* is the width at 0 h and *B* is the width at 24 h [29].

### 2.6. Colony Formation Assay

Cells (8 × 10^2^/well) were cultured in 6-well plates for 2 weeks at 37°C. At room temperature, colonies were fixed with methanol for 15 min and subsequently stained with 0.5% crystal violet for 30 min. The plates were scanned and the numbers of colonies were counted [31].

### 2.7. Soft Agar Assay

Cells (1 × 10^4^/well) were suspended in the medium containing 0.6% agarose and overlaid onto a basal layer of 1.2% agarose in 6-well plates. After 14 days, colonies were observed under an Olympus CKX41 microscope and the numbers of colonies were counted [31].

### 2.8. Migration and Invasion Assays

Migration and invasion assays were performed as previously described [29]. The numbers of stained cells were counted using a Zeiss Axioskop 2 plus microscope (Carl Zeiss, Thornwood, NY, USA).

### 2.9. Detection of Intracellular Reactive Oxygen Species (ROS)

Intracellular ROS were detected by using a Dihydroethidium (DHE) Cellular ROS Detection Assay Kit (Vigorous Biotechnology, Beijing, China).

### 2.10. Western Blotting

After treatment for 24 h, total protein was extracted from TPC-1, TT, and ARO cells. Western blotting was performed to detect the expression of target proteins. The primary antibodies, including anti-*β*-actin, anti-CSE, anti-CBS, anti-3-MST, antisulfide-quinone reductase (SQR), antithiosulfate sulfurtransferase (TST), antiphosphatidylinositol 3-kinase (PI3K), anti-phospho (p)-PI3K (Tyr458/Tyr199), anti-AKT, anti-p-AKT (Ser473), antimammalian target of rapamycin (mTOR), anti-p-mTOR (Ser2448), anti-H-RAS, anti-RAF, anti-p-c-RAF (Ser259), anti-MEK1/2, anti-p-MEK1/2 (Ser217/221), antiextracellular signal-regulated protein kinase 1/2 (ERK1/2), anti-p-ERK1/2 (Thr202/Tyr204), and the horseradish peroxidase-conjugated secondary antibody were purchased from Cell Signaling Technology (CST, Danvers, MA, USA). The results were normalized to the level of *β*-actin. The reaction was visualized using an enhanced chemiluminescence system (Thermo Fisher Scientific, Rockford, IL, USA). The bands were semiquantified with ImageJ software.

### 2.11. Animal Study

Animal experiments were approved by the Committee of Medical Ethics and Welfare for Experimental Animals of Henan University School of Medicine (HUSOM-2017-189) in compliance with the Experimental Animal Regulations formulated by the National Science and Technology Commission, China. Animal studies were conducted as previously described with slight modifications [32]. Forty-eight 4-week-old male BALB/C nude mice (*n* = 8 per group) were purchased from Beijing HFK Bioscience Co. Ltd. (Certificate No. SCXK (Jing) 2014-0004, Beijing, China). TPC-1, TT, and ARO cells (5 × 10^6^ cells in 200 *μ*l PBS) were implanted by subcutaneous injection into the right flanks of mice. Then, the mice were randomly divided into six groups (*n* = 8 per group). NaHS (0.56, 1.4, 2.8, 5.6, and 11.2 mg/kg/day) was administered subcutaneously (near the implanted tumor) for 4 weeks. The control group was treated with PBS. Body weight and tumor volume were daily measured. Tumor volume was calculated as volume = *L* × *W*^2^/2, where *L* is the longest dimension and *W* is the dimension perpendicular to *L* [27]. The tumor volume doubling time (TVDT) was calculated as TVDT = log2/log(*V*2/*V*1) × (*T* – *T*_0_), where *V*2 and *V*1 are tumor volumes and (*T* – *T*_0_) is the time interval [33]. Then, the mice were sacrificed and tumors were excised and weighted. The inhibition rate (IR) of tumor growth was calculated as IR = [(*A* − *B*)/*A*] × 100%, where *A* is the average tumor weight of the control group and *B* is that of the treatment group [32].

### 2.12. Hematoxylin and Eosin (HE) Staining

Tumor samples were fixed in 10% neutral buffered formalin and embedded in paraffin. The specimens were sectioned at 5 *μ*m thickness and processed according to HE staining protocols [31]. Tumor tissues were observed using a Zeiss Axioskop 2 plus microscope.

### 2.13. Immunohistochemistry (IHC) and Evaluation

Tumor samples were stained with anti-Ki67 antibody (CST, Danvers, MA, USA). Ki67-positive tumor cells were observed with a Zeiss Axioskop 2 plus microscope. The proliferation index (PI) was determined as the ratio of the number of Ki67-positive cells to the total number of counted tumor cells [34]. Cluster of differentiation 31 (CD31) is a key biomarker for vascular endothelial cells, and its immunostaining density is represented by the tumor microvessel density (MVD) [35]. Tumor tissues were stained with CD31 antibody (CST, Danvers, MA, USA). Vessels were observed and counted by using a Zeiss Axioskop 2 plus microscope [31].

### 2.14. Statistical Analysis

Data are presented as mean ± standard error of the mean (SEM). The differences between multiple groups were analyzed by one-way analysis of variance using SPSS 17.0 software, followed by Tukey's test. A *P* value of less than 0.05 was considered to be statistically significant.

## 3. Results

### 3.1. Exogenous H_2_S Regulates the Proliferation, Viability, Migration, and Invasion of Human Thyroid Carcinoma Cells

**Figure 1:**
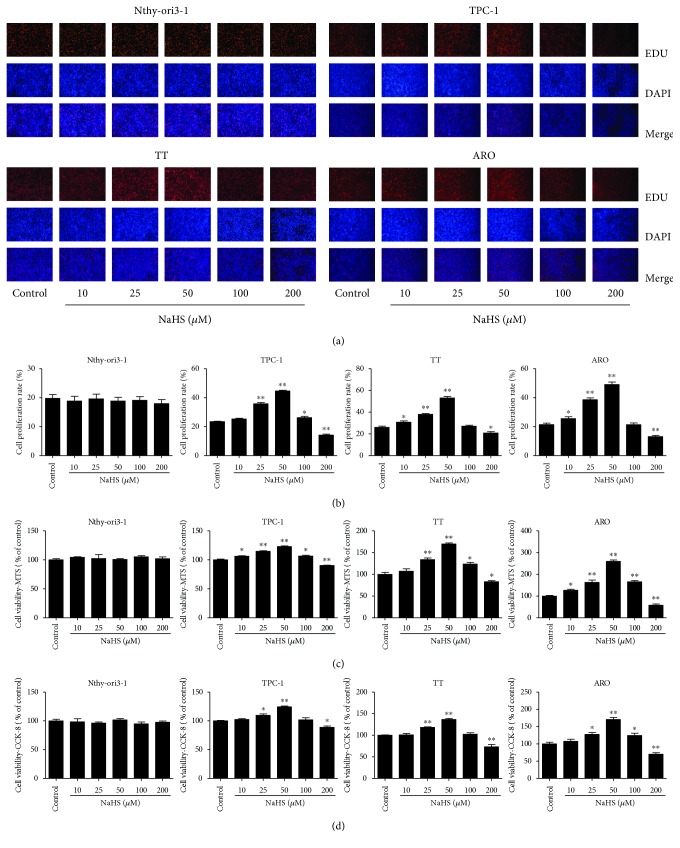
Effects of exogenous H_2_S on the proliferation and viability of human thyroid carcinoma cells and human normal thyroid cells. (a) DNA replication activities of Nthy-ori3-1, TPC-1, TT, and ARO in each group were examined by EdU assay; original magnification ×200. (b) The proliferation rate of each group was analyzed (*n* = 6). (c) The percentages of viable cells were determined using the MTS assay, and the cell viability of every cell line without NaHS treatment was normalized as 100% and considered to be the control group (*n* = 3). (d) The percentages of viable cells were determined using the CCK-8 assay, and the cell viability of every cell line without NaHS treatment was normalized as 100% and considered to be the control group (*n* = 3). ^∗^*P* < 0.05, ^∗∗^*P* < 0.01 compared with the control group.

**Figure 2:**
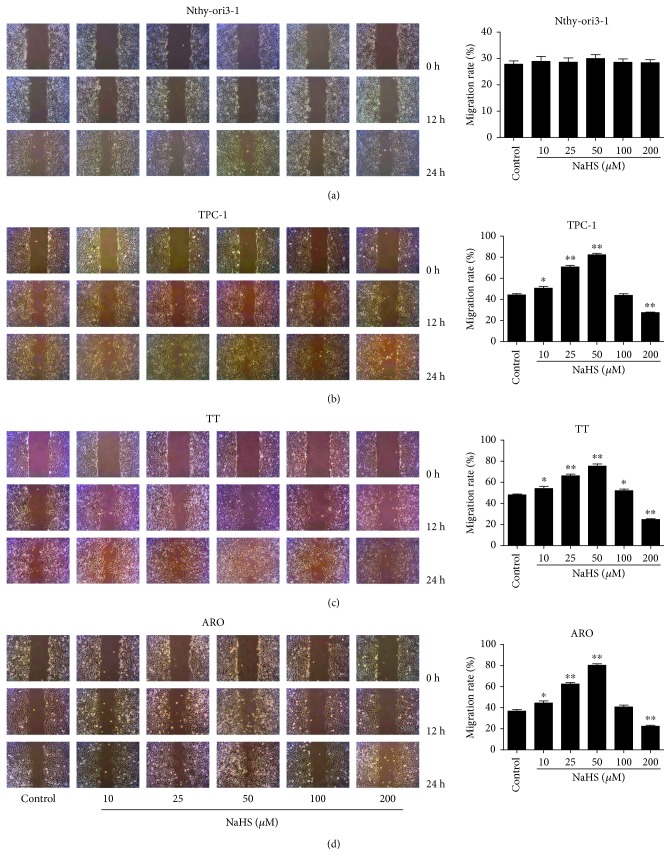
Effects of exogenous H_2_S on the migration of human thyroid carcinoma cells and human normal thyroid cells. (a–d) Cell migration was measured by wound healing assay (original magnification ×100), and the migration rates of Nthy-ori3-1, TPC-1, TT, and ARO cells were calculated after treatment for 24 h (*n* = 6). ^∗^*P* < 0.05, ^∗∗^*P* < 0.01 compared with the control group.

**Figure 3:**
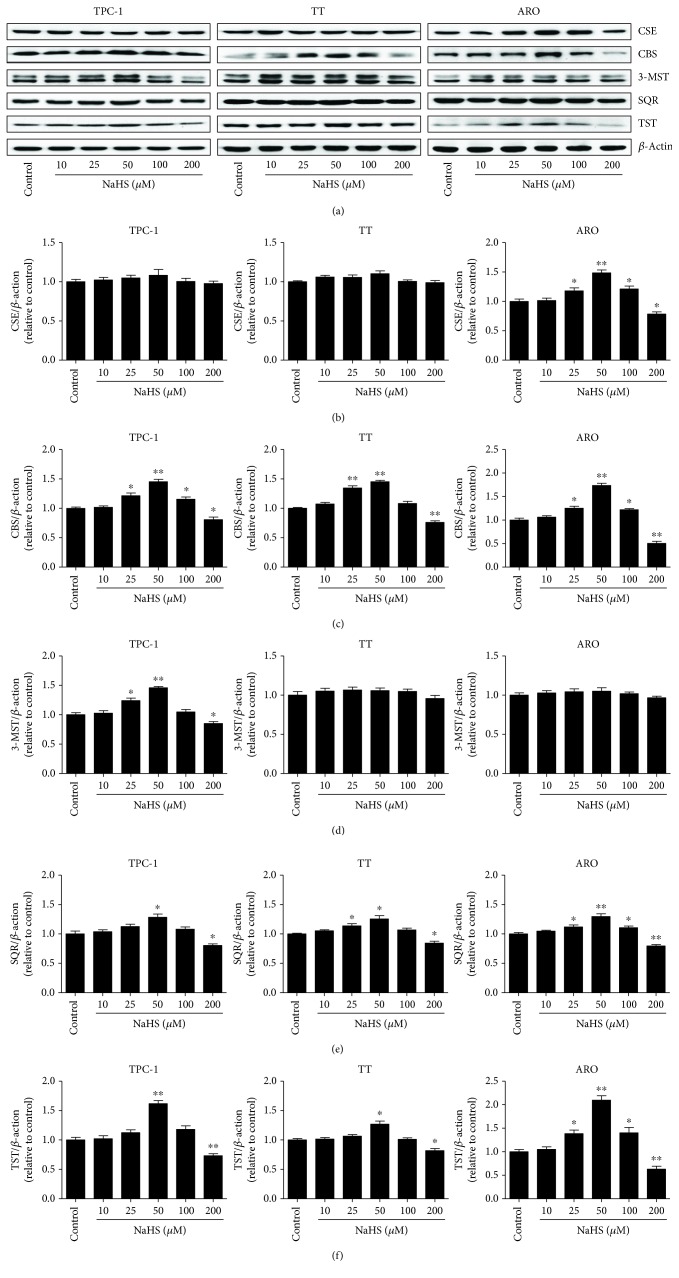
Effects of exogenous H_2_S on the protein levels of H_2_S-generating enzymes and H_2_S-degradating enzymes in human thyroid carcinoma cells. (a) Western blotting analysis of the expressions of CSE, CBS, 3-MST, SQR, and TST in TPC-1, TT, and ARO cells. *β*-Actin was used as the loading control. (b–f) The intensities of the bands were quantified by densitometry analyses and normalized by the amount of *β*-actin (*n* = 3). ^∗^*P* < 0.05, ^∗∗^*P* < 0.01 compared with the control group.

**Figure 4:**
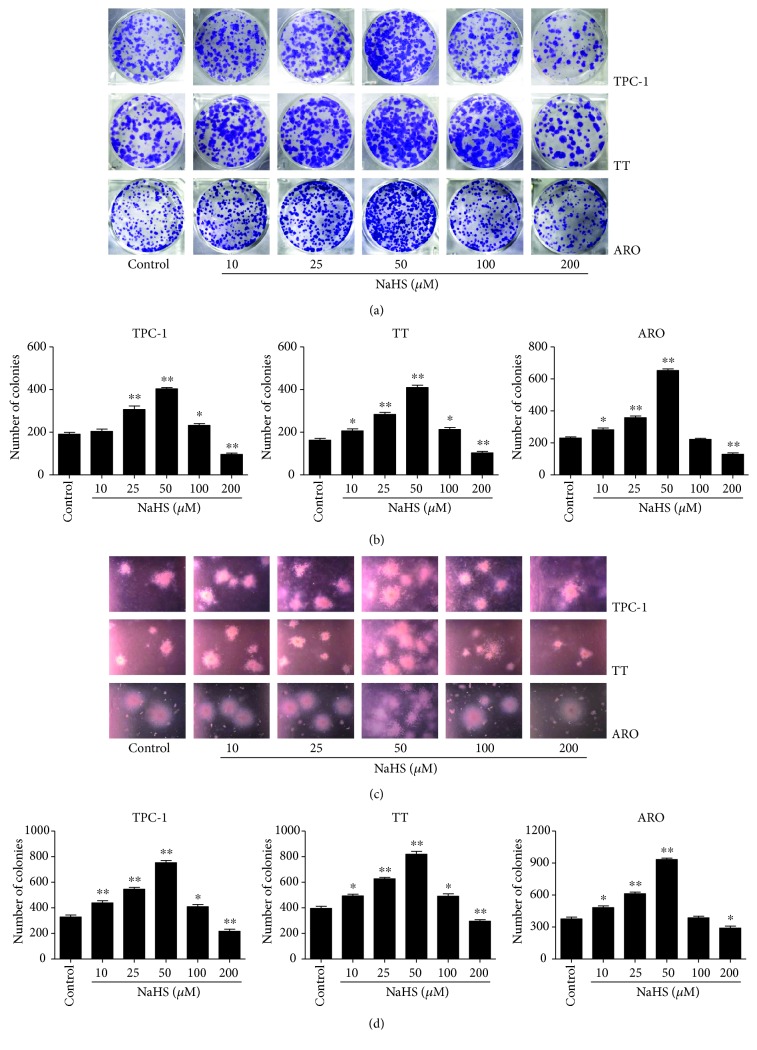
Effects of exogenous H_2_S on the colony formation ability of human thyroid carcinoma cells. (a) The clonogenic capacity was determined in TPC-1, TT, and ARO cells. (b) The number of colonies was calculated (*n* = 3). (c) Soft agar assay was performed to examine the anchorage-independent survival of cells; original magnification ×100. (d) The number of colonies was calculated (*n* = 3). ^∗^*P* < 0.05, ^∗∗^*P* < 0.01 compared with the control group.

**Figure 5:**
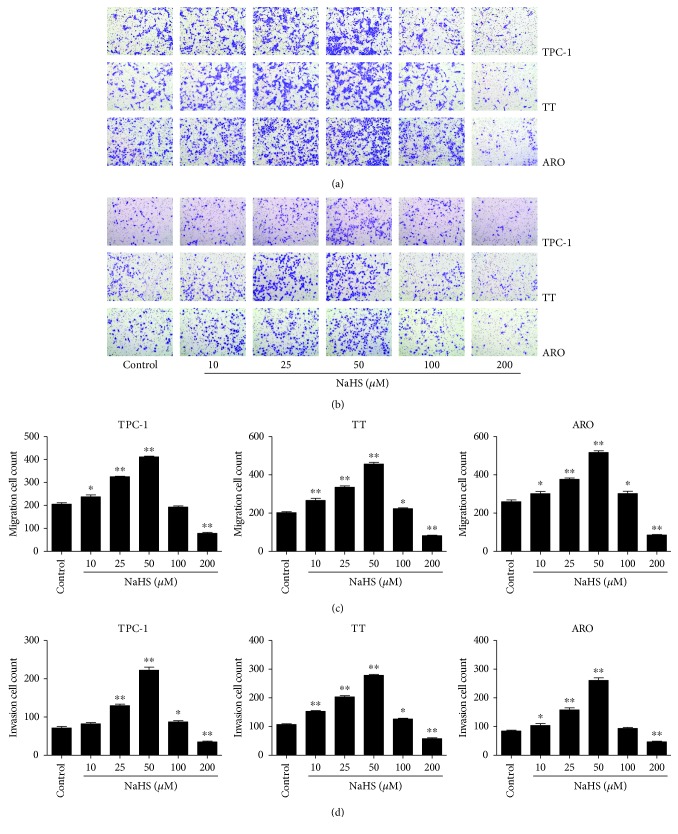
Effects of exogenous H_2_S on the migration and invasion of human thyroid carcinoma cells. (a) Transwell assay was performed to assess the migration of TPC-1, TT, and ARO cells; original magnification ×200. (b) Transwell assay was performed to assess the invasion of TPC-1, TT, and ARO cells; original magnification ×200. (c) The number of the migrated cells was calculated (*n* = 6). (d) The number of the invasive cells was calculated (*n* = 6). ^∗^*P* < 0.05, ^∗∗^*P* < 0.01 compared with the control group.

As shown in Figure 1, the proliferation and viability of TPC-1, TT, and ARO cells were enhanced by 25–50 *μ*M NaHS and inhibited by 200 *μ*M NaHS. However, H_2_S had no obvious effects on the proliferation and viability of Nthy-ori3-1 cells. In addition, 10–50 *μ*M NaHS increased the migration capabilities of human thyroid carcinoma cells and 200 *μ*M NaHS exhibited reverse trends. H_2_S did not affect the migration of human normal thyroid cells (Figure 2). These results indicate that H_2_S is involved in the growth of human thyroid carcinoma cells. The protein levels of H_2_S-generating enzymes and H_2_S-degradating enzymes were further determined. The results showed that 25–50 *μ*M NaHS increased the expression levels of CBS, while 200 *μ*M NaHS exerted reverse effects. Furthermore, 50 *μ*M NaHS increased the expression levels of SQR and TST, whereas 200 *μ*M NaHS showed reverse trends (Figure 3). Moreover, 25–50 *μ*M NaHS increased the number of colonies, while 200 *μ*M NaHS exerted reverse effects (Figure 4). Transwell analysis showed that 10–50 *μ*M NaHS improved the migration capacities and 25–50 *μ*M NaHS increased the invasion capacities of human thyroid carcinoma cells. Treatment with 200 *μ*M NaHS inhibited the migration and invasion capacities of human thyroid carcinoma cells (Figure 5). These results suggest that exogenous H_2_S plays an important role in regulating the proliferation, viability, migration, and invasion of human thyroid carcinoma cells.

### 3.2. Exogenous H_2_S Mediates the ROS/PI3K/AKT/mTOR Signaling Pathway in Human Thyroid Carcinoma Cells

**Figure 6:**
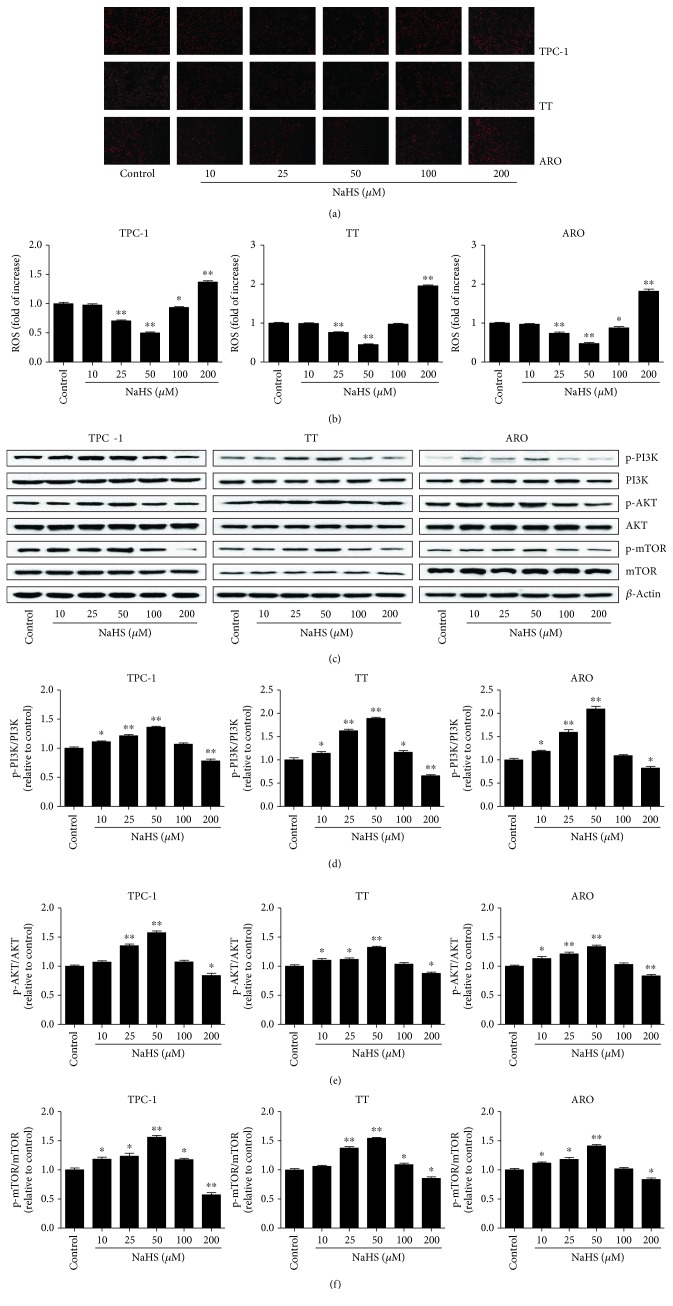
Effects of exogenous H_2_S on the ROS/PI3K/Akt/mTOR signaling pathway in human thyroid carcinoma cells. (a) The intracellular ROS production was detected using the fluorescent probes DHE (shown in red; original magnification, ×100). (b) The intracellular ROS production was measured (*n* = 6). (c) Western blotting analysis of the expressions of PI3K, p-PI3K, AKT, p-AKT, mTOR, and p-mTOR in TPC-1, TT, and ARO cells. *β*-Actin was used as the loading control. (d–f) The intensities of the bands were quantified by densitometry analyses and normalized by the amount of PI3K, AKT, and mTOR, respectively (*n* = 3). ^∗^*P* < 0.05, ^∗∗^*P* < 0.01 compared with the control group.

The PI3K/AKT/mTOR signaling pathway is a critical intracellular signaling cascade involved in a number of hallmarks of cancer, such as cell proliferation, survival, growth, motility, and metabolism [36, 37]. This pathway also plays a key role in many cancer-promoting aspects of the tumor environment, including angiogenesis and inflammatory cell recruitment [37]. Furthermore, it has been demonstrated that PI3K/Akt/mTOR cascade can be driven by ROS [38]. As shown in Figure 6, 25–50 *μ*M NaHS decreased ROS levels and increased phosphorylations of PI3K, AKT, and mTOR, while 200 *μ*M NaHS showed opposite effects, indicating that exogenous H_2_S regulates the proliferation, viability, migration, and invasion of human thyroid carcinoma cells through the ROS/PI3K/AKT/mTOR signaling pathway.

### 3.3. Exogenous H_2_S Mediates the RAS/RAF/MEK/ERK Signaling Pathway in Human Thyroid Carcinoma Cells

**Figure 7:**
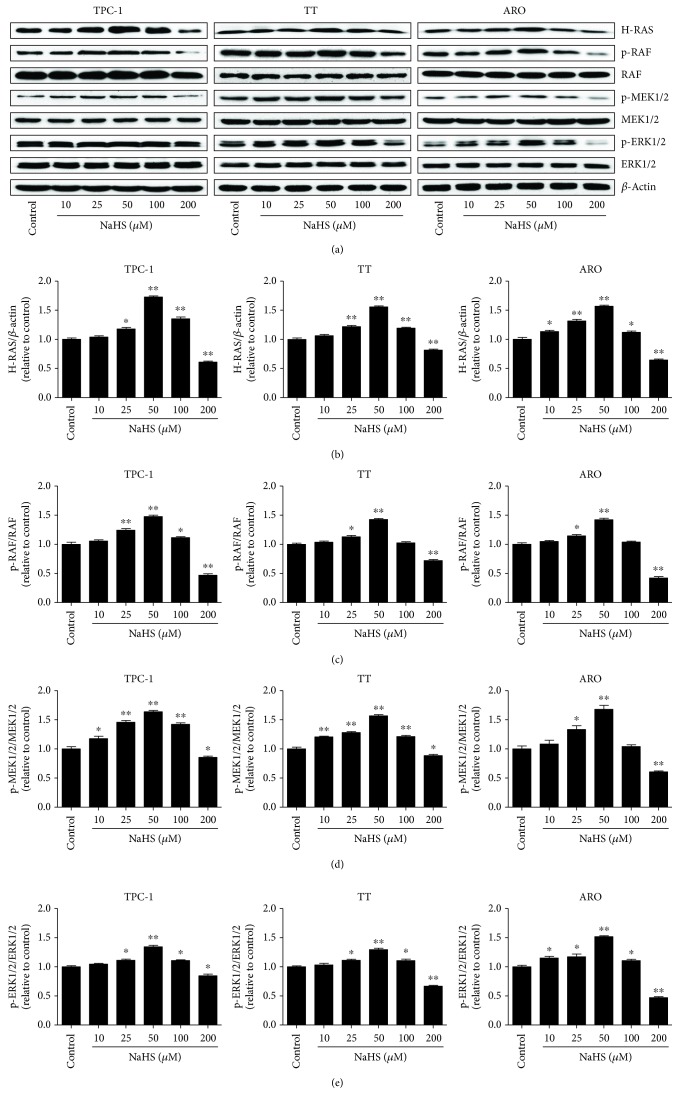
Effects of exogenous H_2_S on the RAS/RAF/MEK/ERK signaling pathway in human thyroid carcinoma cells. (a) Western blotting analysis of the expressions of H-RAS, RAF, p-RAF, MEK1/2, p-MEK1/2, ERK1/2, and p-ERK1/2 in TPC-1, TT, and ARO cells. *β*-Actin was used as the loading control. (b–e) The intensities of the bands were quantified by densitometry analyses and normalized by the amount of *β*-actin, RAF, MEK1/2, and ERK1/2, respectively (*n* = 3). ^∗^*P* < 0.05, ^∗∗^*P* < 0.01 compared with the control group.

The RAS/RAF/MEK/ERK cascade is a key intracellular signaling pathway that regulates many physiological functions and cellular processes, including proliferation, apoptosis, survival, motility, differentiation, and metabolism [39–41]. Hyperactivation of the RAS/RAF/MEK/ERK signaling pathway has been regarded as a hallmark for driving tumorigenesis in a high percentage of human cancers [42, 43]. As shown in Figure 7, treatment with 25–50 *μ*M NaHS increased the protein levels of H-RAS, p-RAF, p-MEK1/2, and p-ERK1/2. However, administration of 200 *μ*M NaHS decreased the expression levels of these proteins. The results suggest that exogenous H_2_S could regulate the proliferation, viability, migration, and invasion of human thyroid carcinoma cells via the RAS/RAF/MEK/ERK signaling pathway.

### 3.4. Exogenous H_2_S Regulates the Growth and Angiogenesis of Human Thyroid Carcinoma Xenograft Tumors in Nude Mice

**Figure 8:**
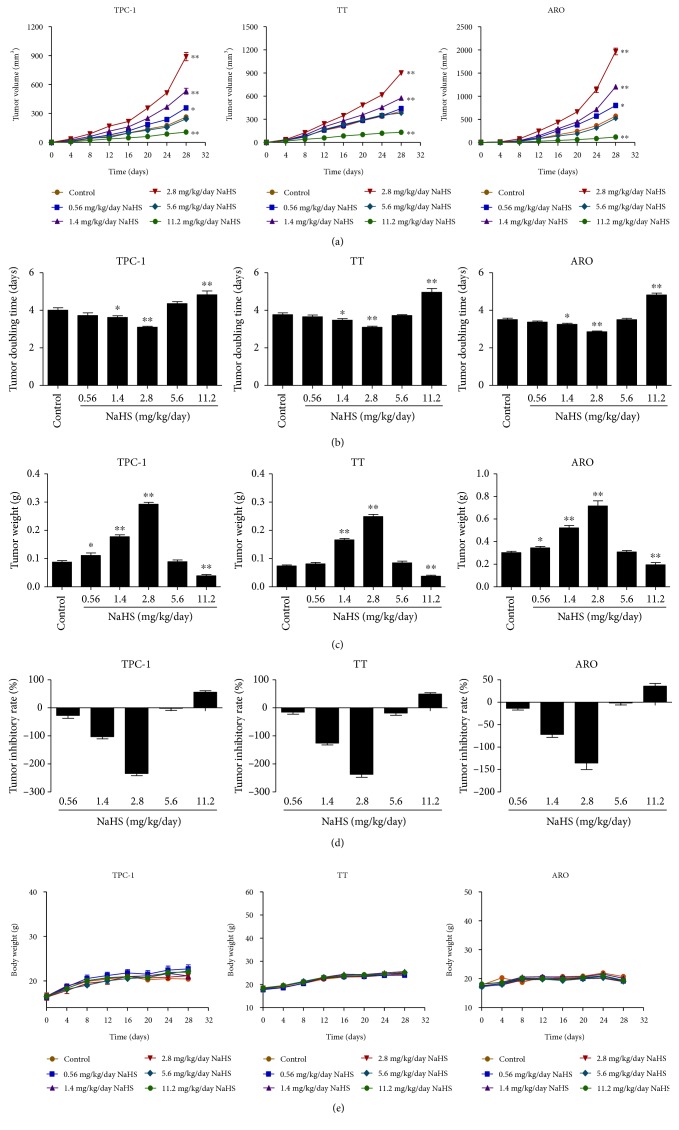
Effects of exogenous H_2_S on the growth of human thyroid carcinoma xenograft tumors in nude mice. (a, b) The tumor volumes of TPC-1, TT, and ARO xenograft tumors were measured every day and the TVDT was calculated by the formula shown above (*n* = 8). (c, d) The tumors were weighed and the inhibition rates of tumor growth were calculated (*n* = 8). (e) The body weight change curve of each group during the experiment (*n* = 8). ^∗^*P* < 0.05, ^∗∗^*P* < 0.01 compared with the control group.

**Figure 9:**
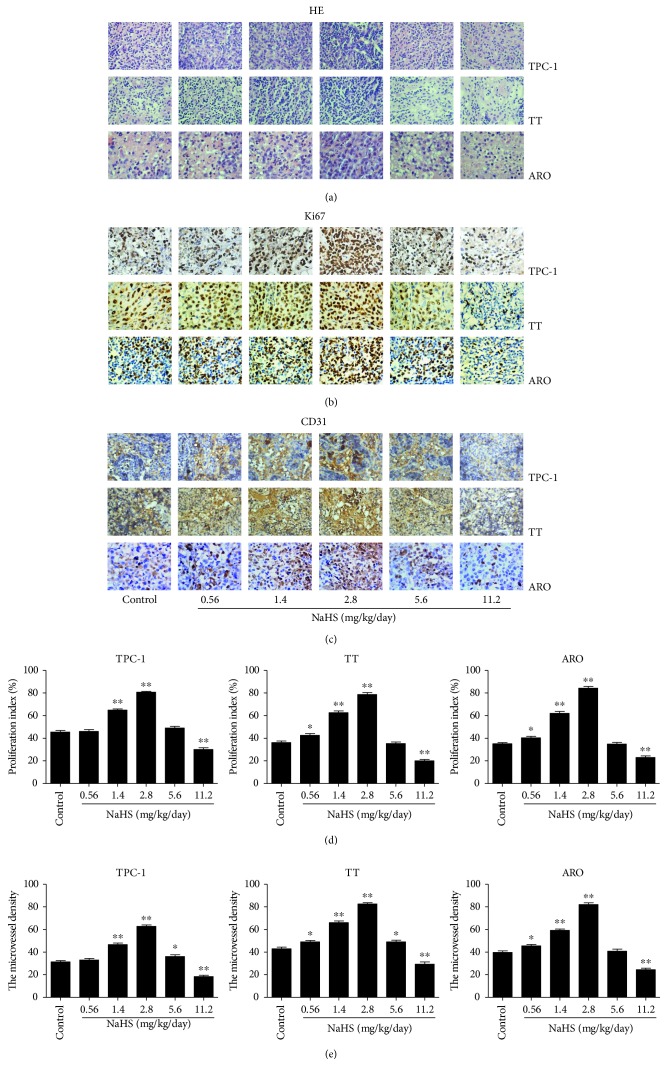
Effects of exogenous H_2_S on the PI and MVD of human thyroid carcinoma xenograft tumors. (a–c) Representative photographs of HE, Ki67, and CD31 staining in TPC-1, TT, and ARO xenograft tumors; original magnification ×400. (d, e) The PI and MVD were calculated (*n* = 6). ^∗^*P* < 0.05, ^∗∗^*P* < 0.01 compared with the control group.

TPC-1, TT, and ARO cells have been successfully used to establish mouse tumor models in cancer research [44–46]. We therefore examined the effect of exogenous H_2_S on the growth of human thyroid carcinoma xenograft in nude mice. Compared with the control group, treatment with 1.4-2.8 mg/kg/day NaHS promoted the growth of xenograft tumors, while administration of 11.2 mg/kg/day NaHS showed opposite effects (Figures 8(a)–(d)). However, there was no significant difference in body weight between each group (Figure 8(e)). IHC with the Ki67 antibody confirmed that the *in vivo* proliferation of human thyroid carcinoma cells was increased by treatment with 1.4-2.8 mg/kg/day NaHS and decreased by treatment with 11.2 mg/kg/day NaHS. Furthermore, the expression level of CD31 in human thyroid carcinoma xenograft tumors exhibited a similar trend (Figure 9). These results together suggest that exogenous H_2_S modulates the growth and angiogenesis of human thyroid carcinoma xenograft tumors.

## 4. Discussion

H_2_S has been considered the third gaseous signaling molecule and plays important roles in the progression of many types of cancer [5, 26–28]. Thyroid cancer is one of the most common endocrine-related cancers with a rapid worldwide rise in incidence in the past few decades [24]. A recent study indicates that diallyl sulfide (an H_2_S donor) could decrease cell proliferation and induce apoptosis via mitochondrial signaling pathway in anaplastic thyroid carcinoma cells [47]. However, whether H_2_S is involved in the growth of human thyroid carcinoma cells remains unknown. Human thyroid carcinoma cell lines TPC-1, TT, and ARO cells have been widely implicated in establishing tumor-bearing animal models [44–46]. In this study, TPC-1, TT, and ARO cells were used to evaluate the effects of exogenous H_2_S both *in vitro* and *in vivo*. The results showed that administration of 25-50 *μ*M NaHS promoted the proliferation and viability, as well as increased the migration and invasion capabilities of TPC-1, TT, and ARO cells when compared with the control group, whereas treatment with 200 *μ*M NaHS exhibited completely opposite effects. However, H_2_S had no obvious effects on the proliferation, viability, and migration of Nthy-ori3-1 cells. In addition, 25–50 *μ*M NaHS increased the expression levels of CBS, while 200 *μ*M NaHS exerted reverse effects, suggesting that CBS may mediate the effects of H_2_S on the growth of human thyroid carcinoma cells. Knockout or knockdown experiments could be performed to clarify the mechanism of action of CBS in the procession of thyroid carcinoma. Treatment with 50 *μ*M NaHS increased the expression levels of SQR and TST, whereas 200 *μ*M NaHS showed reverse trends. These results together suggest that exogenous H_2_S plays important roles in the proliferation, viability, migration, and invasion of human thyroid carcinoma cells. Whether CBS-derived H_2_S could mediate the growth of human thyroid carcinoma cells needs to be further investigated.

The PI3K/AKT/mTOR signaling pathway is involved in cell growth, survival, metabolism, motility, and angiogenesis [48, 49]. PI3K activates the threonine/serine kinase AKT, which could phosphorylate and activate mTOR via a cascade of regulators [48]. The PI3K/AKT/mTOR pathway is one of the most frequently dysregulated pathways in tumor progression, which makes this pathway an attractive target for cancer therapy [48, 50, 51]. It has been reported that ROS could serve as upstream regulators of PI3K, Akt, and mTOR [38, 52, 53]. Furthermore, intracellular ROS play vital roles in PI3K/AKT/mTOR inactivation in human THP-1 monocytes [54] and human prostate cancer cells [55]. A recent study indicates that AKT activity is upregulated in aggressive thyroid cancers where it promotes proliferation and invasion [56]. Furthermore, melanoregulin has been shown to regulate the invasion and proliferation of thyroid cancer cells via PI3K/AKT/mTOR pathway [57]. Another study suggests that 0.1 mM Na_2_S (an H_2_S donor) decreases ROS formation, however, 0.5 mM Na_2_S induces an increase in ROS formation in HeLa cells, indicating that relatively low doses of H_2_S can inhibit the oxidative stress and relatively high levels of H_2_S show opposite effects [58]. In addition, H_2_S has exerted multiple biological effects on HCC cells by inhibiting the PI3K/Akt/mTOR pathway [59]. Our results demonstrated that 25–50 *μ*M NaHS promoted the proliferation, viability, migration, and invasion of human thyroid carcinoma cells by downregulating ROS levels and upregulating phosphorylations of PI3K, AKT, and mTOR. However, administration of 200 *μ*M NaHS decreased the expression levels of these proteins. The results indicate that exogenous H_2_S regulates the growth of human thyroid carcinoma cells through the ROS/PI3K/AKT/mTOR signaling pathway.

The RAS/RAF/MEK/ERK pathway comprises three dual-specific protein kinases RAF, MEK, ERK, and the G-protein RAS [60]. The binding of different ligands to receptor tyrosine kinases at the cell surface can induce the activation of RAS which in turn activates RAF, MEK, and ERK [60, 61]. The activated ERK translocates into the nucleus and activates transcription factors to induce a number of cellular functions [39, 62]. The RAS/RAF/MEK/ERK pathway plays key roles in cancer development, maintenance, and progression, which may contribute to drug resistance and poorer prognosis [63]. It has been shown that the RAS/RAF/MEK/ERK pathway is one of the most frequently activated oncogenic signaling pathways in thyroid cancer [64]. A previous study indicates that the majority of differentiated thyroid cancer patients exhibit a detectable genetic alteration affecting the RAF/MEK/ERK pathway [65]. In addition, diallyl disulfide (DADS) can suppress the growth of human esophageal xenograft tumors through RAF/MEK/ERK and mitochondria-dependent pathways [66]. Considering H_2_S can be released by DADS [67], whether H_2_S mediates the effect of DADS on the growth of human esophageal cancer cells needs to be further investigated. Our results showed that 25–50 *μ*M NaHS increased the protein levels of H-RAS, p-RAF, p-MEK1/2, and p-ERK1/2, while treatment with 200 *μ*M NaHS showed opposite effects. The results suggest that exogenous H_2_S regulates the proliferation, viability, migration, and invasion of human thyroid carcinoma cells via the RAS/RAF/MEK/ERK signaling pathway.

TPC-1, TT, and ARO cells have been widely adopted to establish subcutaneous xenograft models [44–46]. We therefore examined the effect of exogenous H_2_S on the growth of human thyroid carcinoma xenograft tumors in BALB/c nude mice. Compared with the control group, 1.4-2.8 mg/kg/day NaHS promoted the growth of xenograft tumors, while administration of 11.2 mg/kg/day NaHS exhibited the inhibitory effects on the growth of xenograft tumors. Ki67 is a key proliferative marker and has been widely used in detecting the proliferation of malignant cells [34, 68]. The results indicated that the expression of Ki67 was increased by treatment with 1.4-2.8 mg/kg/day NaHS and decreased by treatment with 11.2 mg/kg/day NaHS, which were in agreement with the findings *in vitro*. CD31 has been regarded as an ideal biomarker for vascular endothelial cells, and its density is represented by the tumor MVD [35, 69]. The protein expression of CD31 in each group showed a similar trend. In sum, these results indicate that exogenous H_2_S could modulate the growth and angiogenesis of human thyroid carcinoma xenograft tumors.

In conclusion, our results demonstrate that exogenous H_2_S is able to regulate the growth of human thyroid carcinoma cells both *in vitro* and *in vivo*. Novel H_2_S-releasing donors/drugs can be designed and applied for the treatment of thyroid cancer.

## Data Availability

The raw data used to support the findings of this study are available from the corresponding authors upon request.
